# Pathogenicity and epidemiological survey of fowl adenovirus in Shandong Province from 2021 to 2022

**DOI:** 10.3389/fmicb.2023.1166078

**Published:** 2023-05-10

**Authors:** Tailong Wang, Fanliang Meng, Changxiu Chen, Yesheng Shen, Peixun Li, Jie Xu, Zhaoyang Feng, Xiuchao Qu, Fuyong Wang, Baoquan Li, Mengda Liu

**Affiliations:** ^1^Shandong Provincial Key Laboratory of Animal Biotechnology and Disease Control and Prevention, College of Animal Science and Veterinary Medicine, Shandong Agricultural University, Tai'an, China; ^2^Veterinary Clinical Laboratory, College of Agricultural and Forestry Sciences, Linyi University, Linyi, China; ^3^Division of Zoonoses Surveillance, China Animal Health and Epidemiology Center, Qingdao, China

**Keywords:** fowl adenovirus, *hexon* gene, genetic evolutionary analysis, epidemiological survey, pathogenicity

## Abstract

In recent years, the poultry industry had been markedly affected by adenoviral diseases such as hydropericardium syndrome and inclusion body hepatitis caused by fowl adenovirus (FAdV), which have become increasingly prevalent in China. Shandong Province, China, is an important area for poultry breeding where various complex and diverse FAdV serotypes were isolated. However, the dominant strains and their pathogenic characteristics are not yet reported. Therefore, a pathogenicity and epidemiological survey of FAdV was conducted, showing that the local dominant serotypes of FAdV epidemics were FAdV-2, FAdV-4, FAdV-8b, and FAdV-11. Their mortality rates in the 17-day-old specific-pathogen-free (SPF) chicks ranged from 10 to 80%; clinical signs included mental depression, diarrhea, and wasting. The maximum duration of viral shedding was 14 days. The highest incidence in all infected groups was on days 5–9, and then gradual regression occurred thereafter. The most pronounced symptoms occurred in chicks infected with FAdV-4, including pericardial effusion and inclusion body hepatitis lesions. Our results add to the current epidemiological data on FAdV in poultry flocks in Shandong and elucidate the pathogenicity of dominant serotypes. This information may be important for FAdV vaccine development and comprehensive epidemic prevention and control.

## 1. Introduction

Since the first discovery of chicken inclusion body hepatitis (IBH) in the USA in 1963, the disease has become prevalent in many countries worldwide and has markedly affected the global poultry industry (Guan et al., 2018). IBH is caused by fowl adenoviruses (FAdVs) that exhibit diverse pathogenicity. Most infections are subclinical and consistently lead to infections throughout the farm (Schachner et al., 2018). FAdVs can cause growth retardation, IBH, hydropericardium syndrome (HPS), adenoviral gizzard erosion (AGE), decreased egg production, and immunosuppression (Shah et al., 2017; El-Shall et al., 2022), and infections may result in 10–80% mortality (Ganesh and Raghavan, 2000).

FAdV belongs to the genus *Adenovirus* in the family *Adenoviridae*, and it is an icosahedral structured virus with a nucleocapsid containing *hexon, penton*, and *fiber* (Bertran et al., 2021). The *hexon*, as the major capsid protein, is the main antigenic determinant of virus type, group, and subgroup, and its nucleotide sequence is a reliable marker for phylogenetic genotyping (Choi et al., 2012). Based on the *hexon* gene, FAdV can be classified into 12 serotypes, including FAdV-1 to FAdV-7, FAdV-8a, FAdV-8b, and FAdV-9 to FAdV-11. Five species can be classified according to the molecular structural characteristics of the virus, i.e., A (FAdV-1), B (FAdV-5), C (FAdV-4 and FAdV-10), D (FAdV-2, FAdV-3, FAdV-9, and FAdV-11), and E (FAdV-6, FAdV-7, FAdV-8a, and FAdV-8b) (Li et al., 2016a; Du et al., 2017; Niu et al., 2018; Mirzazadeh et al., 2020). Typically, FAdV-1 can cause AGE, FAdV-4 can cause HPS, and FAdV-11 can cause IBH (Marek et al., 2010; Schachner et al., 2018). FAdV-4 was reported as the predominant serotype in China after the HPS outbreak in 2014; however, its prevalence gradually decreased since 2016 with the widespread use of the FAdV-4 inactivated vaccine. By 2020, FAdV-4, FAdV-8a, FAdV-8b, and FAdV-11 were prevalent to varying degrees (Li et al., 2016a). FAdV-4 vaccine immunization has become common practice in various large-scale farms; however, monovalent vaccines exhibit poor cross-protection between different serotypes (Kim et al., 2014). Thus, the development of efficient polyvalent vaccines is a matter of urgency, considering the current prevalence of multiple serotypes (Echavarría, 2008).

Shandong Province, China, is an important area for poultry breeding where various complex and diverse FAdV serotypes have been found. In this study, we examined the currently predominant FAdV serotypes in Shandong Province by conducting an epidemiological survey. In addition, few comparative pathological studies of prevalent FAdV strains have been conducted (Niu et al., 2022). Thus, we performed animal experiments to analyze the pathogenicity of the various serotypes in order to understand the pathogenic characteristics of the currently prevalent strains.

## 2. Materials and methods

### 2.1. Collection of samples

Liver samples were collected from broilers or laying hens suspected to be infected with FAdV. The samples were collected from 2021 to 2022 throughout Shandong Province.

### 2.2. Extraction and PCR identification of samples

The tissue samples were ground to homogenates using a high-throughput tissue grinder, followed by centrifugation at 12,000 rpm/min for 10 min. The supernatant was repeatedly frozen and thawed three times. Genomic nucleic acids of the virus were extracted using a Simply P Virus DNA/RNA Extraction Kit (Bioer Technology, Hangzhou, China).

The samples were tested for FAdV, chicken infectious anemia virus (CIAV), hepatitis E virus (HEV), infectious bronchitis virus (IBV), Marek's disease virus (MDV), avian influenza virus (AIV), avian leukosis virus (ALV), and reticuloendotheliosis virus (REV) by PCR/RT-PCR (primer sequences are shown in Table 1). The PCR mixture of 25 μl included 12.5 μl of 2 × Taq PCR Master Mix (AG, AG11112), 1.25 μl of 1 μM forward and reverse primer, each, 3 μl of DNA, and 7 μl of ddH_2_O. The following PCR conditions were used: 94°C for 30 s, followed by 32 cycles of 98°C for 10 s, 55°C for 30 s, 72°C for 1 min, and a final step of 72°C for 2 min. The RT-PCR mixture of 25 μl included 12.5 μl of 2 × One Step Mix, 1.5 μl of One Step Enzyme mix, 1 μl of 10 μM forward and reverse primer, each, 3 μl of RNA, and 6 μl of ddH_2_O. The following RT-PCR conditions were used: reverse transcription at 50 °C for 30 min and pre-denaturation at 94°C for 3 min; 32 cycles of 94°C for 30 s, 55°C for 30 s, 72°C for 1 min, and a final step of 72°C for 7 min. Then, 10 μl of the PCR products was added to 1% agarose gels, and amplicons were visualized using a gel imaging device.

**Table 1:** Primers for viral nucleic acid detection.

**Viruses**	**Names**	**Primer sequence (5^′^−3^′^)**	**Length (bp)**
FAdV	FAdV-F	AATTTCGACCCCATGACGCGCCAGG	508
	FAdV-R	TGGCGAAAGGCGTACGGAAGTAAGC	
CIAV	CIAV-F	CAGAATTCCCACCTCAAGCGACTTCGAC	582
	CIAV-R	ATGTCGACGGGGCTGAAGGAT	
HEV	HEV-F	ATGGACGTCTCGCAGTTTGCAGAGT	707
	HEV-R	TGTAACACCTCGCGCTTATGCACAT	
IBV	IBV-F	GGTATAGTGTGGGTTGCTG	459
	IBV-R	CCTTAATACCTTCCCATTC	
MDV	MDV-F	TCATCAGGGTCTCCCGTCACCT	1,005
	MDV-R	AGAGATGTCTCAGGAGCCAGAG	
AIV	AIV-F	ATGAGYCTTYTAACCGAGGTCGAA	280
	AIV-F	TGGACAAANCGTCTACGCTGCAG	
ALV	ALV-R	GGATGAGGTGACTAAGA	512
	ALV-R	CGAACCAAAGGTAACACACG	
REV	REV-F	CATACTGAGCCAATGGTT	300
	REV-R	AATGTTGTAGCGAAGTACT	

### 2.3. Virus isolation

FAdV-positive samples were isolated using the LMH cell line (ATCC CRL-2177^TM^). The tissue supernatant, which was stored at −80°C, was repeatedly frozen and thawed three times, centrifuged at 12,000 rpm for 10 min, and was then filtered using a sterile 0.22-μm filter. Then, a 1 ml sample was inoculated in a cell bottle (5 ml) filled with a single layer of LMH, which was sensitized at 37°C and 5% CO_2_ for 2 h. The supernatant was discarded, and 5 ml of Dulbecco's modified Eagle's medium (DMEM) containing 1% fetal bovine serum (FBS; Gibco, San Diego, CA, United States) was added, followed by incubation at 37°C and 5% CO_2_. After 80% of the cells showed lesions, the viral fluid was harvested and repeatedly frozen and thawed three times. After the cell debris was removed by centrifugation, the viral fluid was stored at −80°C.

### 2.4. Sequencing and analysis of the *hexon* gene

PCR amplification of FAdV-positive samples was performed using primers specific to the *hexon* gene (Table 2). In total, 25 reference strains were downloaded from NCBI for comparison with the strains in this study using MEGA 11 and MegAlign (DNA STAR). Maximum-likelihood evolutionary analyses were performed using MEGA 11. Multiple sequence comparisons were generated using Clustal W. ITOL was used to modify the genetic evolutionary tree using different colors to distinguish FAdV species.

**Table 2:** Primer sequences for amplification of the full-length *hexon* genes of various FAdVs.

**Species**	**Names**	**Primer sequence (5'−3')**	**Length (bp)**	**Serotype**
FAdV-A	FAdV-A-F	AGGCTCTCATTCAGGCCC	3,492	1
	FAdV-A-R	GCGAACCCGATCCAGTGC		
FAdV-B	FAdV-B-F	CCACCAGACGCACCAACA	3,311	5
	FAdV-B-R	TCCGAACGGGTCGAACAT		
FAdV-C	FAdV-C-F	GAGATGGTGACGGAGGTG	3,139	4, 10
	FAdV-C-R	AAGCGGTGACGAGGATGC		
FAdV-D	FAdV-D-F	GAGATGGTGACGGAGGTG	3,286	2, 3, 9, 11
	FAdV-D-R	TTGGGATCGAGGAACCCG		
FAdV-E	FAdV-E-F	GCGAAGAGGAGACGAAAGC	3,185	6, 7, 8a, 8b
	FAdV-E-R	CGAACACGCCCAAGAACC		

Primers were used as described by Hou et al. (2021).

### 2.5. Pathogenicity

In total, four representative prevalent strains were purified using an empty spot assay, and their TCID_50_ was determined using the Reed–Muench method. Then, 200 SPF chicks at 17 days of age were randomly assigned to five groups (*N* = 40, each) comprising four infection treatments (with FAdV-2, FAdV-4, FAdV-8b, and FAdV-11) and a control group. The infection groups were administered 0.2 ml virus solution containing 10^6^ TCID_50_ by intramuscular injection in the chest. The control group was injected with 0.2 ml of PBS. Each group was kept separate from the others. Clinical signs and mortality in each group were observed for 14 consecutive days; body weight was recorded daily to assess growth rates, and cloacal swabs were collected to assess viral shedding. Three chickens from each group were randomly selected to be euthanized for necropsy on days 1, 3, 5, 7, 9, 11, and 14 after infection. According to experimental methods (Chen et al., 2020), the distribution of the virus in each tissue, including the heart, liver, spleen, lung, kidneys, and bursa, was detected. The viral load in blood was also recorded to assess the duration of viremia. Dead chickens were necropsied to assess gross pathological lesions. Samples of the heart, liver, spleen, and kidneys were fixed in 10% neutral-buffered formalin for further histology analysis.

### 2.6. Statistical analyses

All data analyses were performed using independent-samples *t*-tests or two-way ANOVA in SPSS 26.0 software (IBM, Armonk, NY, USA).

## 3. Result

### 3.1. Samples

**Figure 1:**
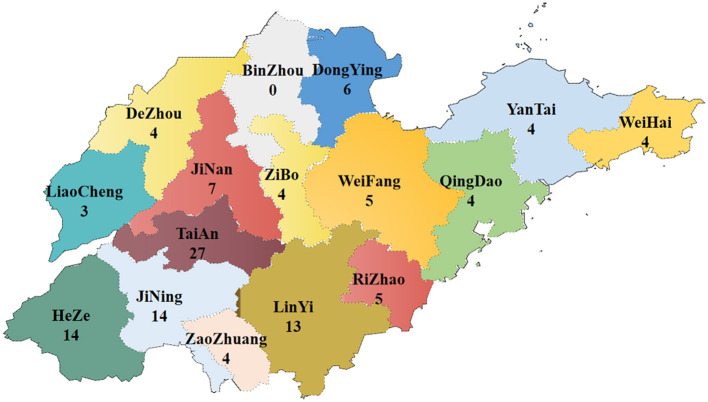
Map of sample collection and distribution in Shandong Province from 2021 to 2022 (numbers indicate the positive samples).

From 2021 to 2022, 601 samples of poultry suspected to be infected with FAdV were collected from various farms distributed in all cities of Shandong Province. In total, 118 FAdV-positive cases were found, with a positive rate of 19.63% (118/601). FAdV was detected in 16 of 17 cities in Shandong (Figure 1).

**Figure 2:**
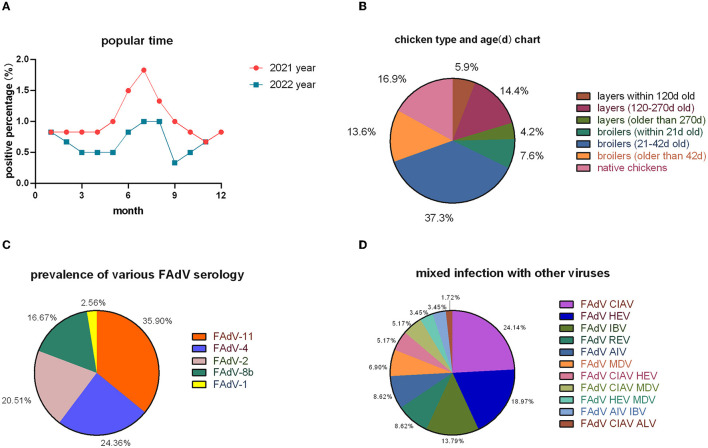
Epidemiological surveys. **(A)** Temporal distribution; **(B)** inter-cluster distribution; **(C)** serotype statistics; and **(D)** mixed infections with other viruses.

The positive rate was lower in 2022 than in 2021 (Figure 2A), but the overall monthly prevalence trend was consistent. In broilers, infections were most common at 21–42 days of age and in laying hens at 120–270 days of age (Figure 2B).

In the 118 positive cases, 78 strains of FAdVs were isolated; 28 strains belonged to FAdV-11 (35.90%), 19 strains to FAdV-4 (24.36%), 16 strains to FAdV-2 (20.51%), 13 strains to FAdV-8b (16.67%), and 2 strains to FAdV-1 (2.56%) (Figure 2C). Co-infection occurred in 58 cases, with FAdV having the highest incidence of co-infection with CIAV and HEV (Figure 2D).

### 3.2. Sequencing and analysis of the *hexon* gene

**Figure 3:**
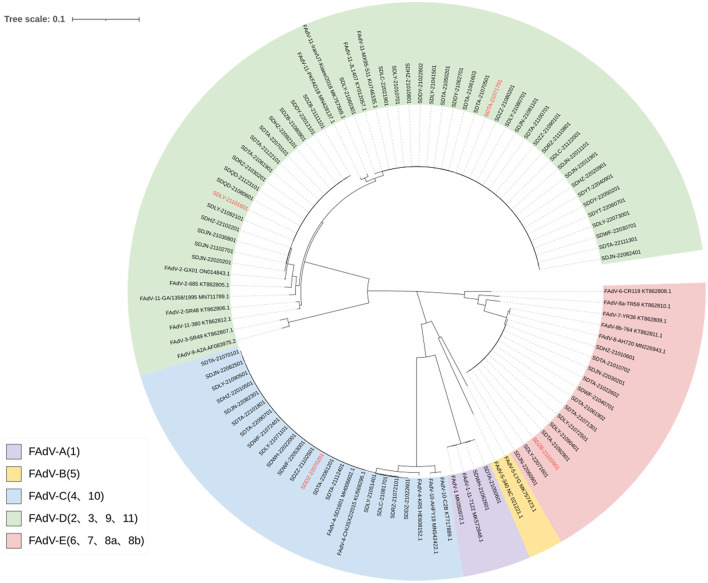
Phylogenetic tree based on FAdV *hexon* gene sequences. The registration number of the reference strain is marked after the strain. The serotype of the reference strain is marked in front of the strain. The strains marked in red are those used for pathogenicity assessment.

After sequencing and splicing, the sequences of 78 FAdVs were submitted to GenBank (GenBank accession numbers: OP917852–OP917924 and OP920975–OP920979). Phylogenetic analyses were performed based on the obtained sequences and the reference sequences downloaded from NCBI. The results are shown in Figure 3. A phylogenetic tree indicated that 44 isolates belonged to FAdV-D (FAdV-2 and FAdV-11), 19 isolates to FAdV-C (FAdV-4), 13 isolates to FAdV-E (FAdV-8b), and two isolates to FAdV-A (FAdV-1).

All FAdV-2 isolates showed 100% homology with each other and 99.61% nucleotide homology with the *hexon* gene sequence of the reference strain GX01 (GenBank accession number: ON014843.1). All FAdV-11 isolates showed 100% homology with each other and with the reference strain MX95-S11 (KU746335.1). All FAdV-4 isolates were 99.9–100% homologous with each other and 99.89–100% with the reference strain SD1601 (MH006602.1). All FAdV-8b isolates were 100% homologous with each other and 99.58–99.96% homologous with the reference strains AH720 (KY968968.1) and 764 (KT862811.1). All FAdV-1 isolates showed 100% homology with each other and 99.93% with the reference strain 11-7127 (MK572848.1).

### 3.3. Pathogenicity

Individuals infected with FAdV-4 and FAdV-8b showed symptoms, such as depression, shrunken head, tousled feathers, and white- or blood-tinged feces 1 day post-infection (dpi), until the clinical symptoms gradually decreased 9 dpi and disappeared 11 dpi. The FAdV-2- and FAdV-11-infected groups exhibited abnormal vocalization and feces with white coloration or blood at 3 dpi, followed by a significant reduction in symptoms at 11 dpi.

**Figure 4:**
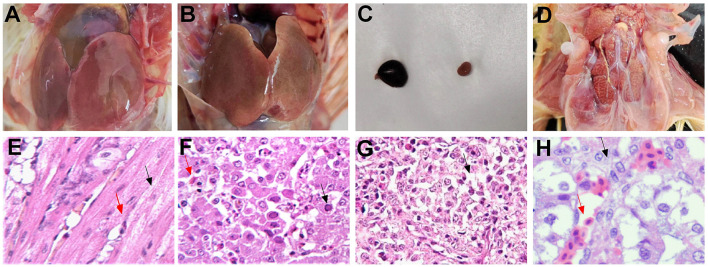
Typical autopsy and microscopic lesions (HE × 200) observed in pathogenicity tests for infection with the SDDZ-22070201 (FAdV-4) strain. **(A)** The heart: pericardial effusion; **(B)** the liver: gray-white necrotic foci and punctate hemorrhage; **(C)** the spleen: enlarged and congested; **(D)** the kidney: pale and swollen; **(E)** the heart: degeneration of myocardial fibrous granules [black arrow in **(E)**] and infiltration of heterophilic granulocytes [red arrow in **(E)**]; **(F)** the liver: hepatic cell necrosis [red arrow in **(F)**] and inclusion body [black arrow in **(F)**]; **(G)** the spleen: loss of lymphocytes [black arrow in **(G)**]; and **(H)** the kidney: tubular epithelial cell degeneration [black arrow in **(H)**] and kidney interstitial congestion [red arrow in **(H)**].

During an autopsy, the FAdV-4 infected group showed the most severe lesion. Severe pericardial effusion was observed (Figure 4A). The liver showed yellow-brown swollen and gray-white necrotic foci and punctate hemorrhage (Figure 4B). The spleen was enlarged and congested (Figure 4C). The kidneys were pale and swollen (Figure 4D). Microscopic observation revealed degeneration of myocardial fibrous granules, interstitial congestion, edema, and infiltration of heterophilic granulocytes (Figure 4E). Hepatic cells were degenerated and necrotic and had numerous basophilic inclusion bodies; the liver was infiltrated and proliferated with a large number of macrophages and lymphocytes (Figure 4F). Congestion, hemorrhage, and loss of lymphocytes were observed in the spleen (Figure 4G). The renal tubular epithelial cells showed degeneration; the kidneys exhibited interstitial congestion and hemorrhage (Figure 4H).

**Figure 5:**
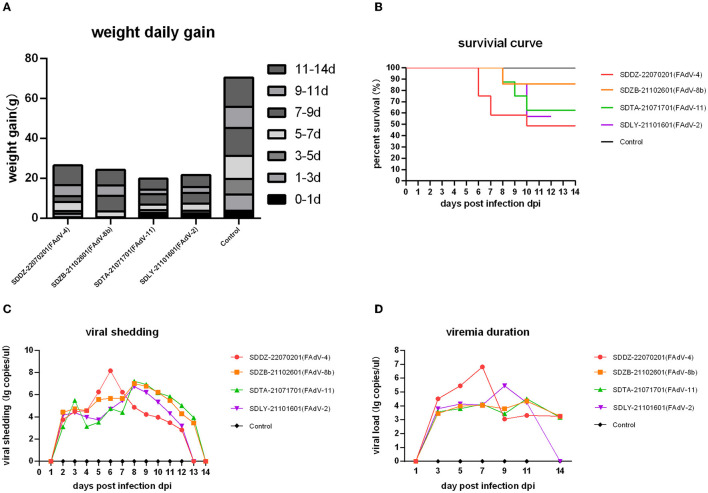
Pathogenicity analysis in chickens infected with various FAdV strains. **(A)** Daily weight gain of infected chickens. **(B)** Survival curve. **(C)** Viral shedding. **(D)** Viremia duration.

Weight gain statistics showed that all four infected groups gained <30 g throughout the trial period, while the control group gained an average of 70 g (Figure 5A), with no significant differences between the infected groups. Death first occurred in the FAdV-4 infected group at 5 dpi, with a final mortality rate of 60%. Deaths in the FAdV-2-, FAdV-8b-, and FAdV-11-infected groups occurred at 8 dpi, with a final mortality rate of 30, 10, and 30%, respectively (Figure 5B).

Cloacal viral shedding of FAdV-2 and FAdV-4 lasted from 1 to 13 dpi and that of FAdV-8b and FAdV-11 from 1 to 14 dpi. In the FAdV-4 infection group, viral shedding peaked at 6 dpi, and in the other three groups at 8 dpi (Figure 5C). FAdV-4, FAdV-8b, and FAdV-11 viremia persisted from 1 to 14 dpi, and FAdV-2 viremia persisted from 1 to 13 dpi. The viral load in blood peaked at 7 dpi in the FAdV-4-infected group, 9 dpi in the FAdV-2-infected group, 11 dpi in the FAdV-8b-infected group, and 11 dpi in the FAdV-11-infected group (Figure 5D).

**Figure 6:**
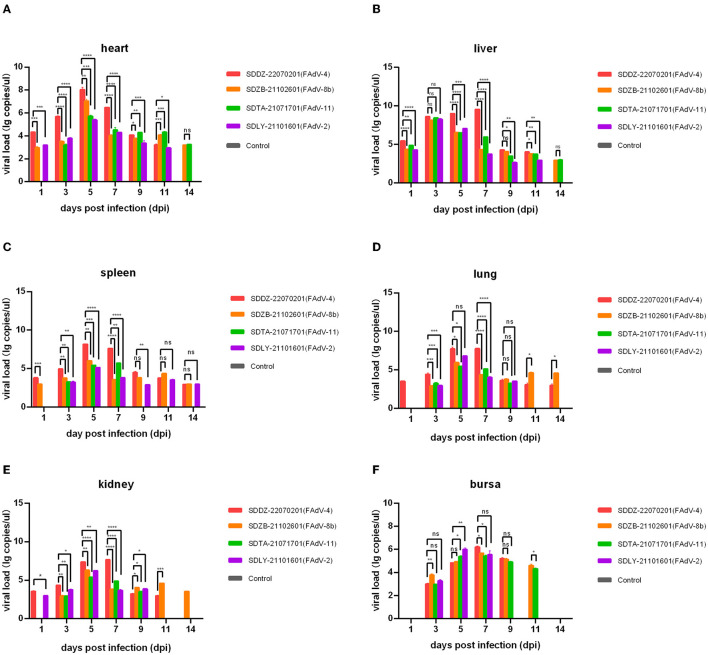
Viral load in various chicken tissues at 1, 3, 5, 7, 9, 11, and 14 days post-infection assessed by qPCR. **(A)** Heart; **(B)** liver; **(C)** spleen; **(D)** lung; **(E)** kidney; **(F)** bursa. Ns indicated no significance, values of *P* < 0.05 were considered statistically significant (*), values of *P* < 0.01 were considered significant differences (**), values of *P* < 0.001 were considered highly significant differences (***), and values of *P* < 0.0001 were considered highly significant (****).

Nucleic acids were extracted from the collected tissues and assayed by qPCR to assess virus distribution (i.e., in the heart, liver, spleen, lung, kidney, and bursa). The growth period of virus replication predominantly occurred at 3–7 dpi, which was when the viral load in each tissue peaked (Figure 6). The FAdV-4 infection group had significantly higher viral loads than the other three infection groups 1, 3, 5, and 7 dpi, except for that in the bursa. The highest viral load among all infection groups was found to occur in the FAdV-4 group with the liver at 5 dpi (10^9.53^ copies/μl).

## 4. Discussion

Since the outbreak of FAdV-4 in China in 2015, HPS has become one of the most severe infectious diseases affecting the chicken industry in China, thus attracting research interest in avian adenovirus diseases (Li et al., 2016b; Niu et al., 2017). Numerous studies have shown that chickens are severely infected with FAdV, and the prevalent FAdV serotypes are complex and diverse (Steer et al., 2015; Li et al., 2022). FAdV-4 has become the dominant serotype in China since 2016; however, owing to the widespread use of the FAdV-4 vaccine, FAdV-4 prevalence has gradually decreased (Li et al., 2016a; Liu et al., 2022). In 2020, serotypes, such as FAdV-4, FAdV-8a, FAdV-8b, and FAdV-11, were prevalent to varying degrees (Steer et al., 2015). Recent epidemiological surveys of FAdV in Shandong Province have not reported relevant details, and there is a lack of data to support the development of preventive and control measures for avian adenovirus diseases.

In the present study, 601 cases of suspected avian adenovirus disease in Shandong Province were analyzed, 118 positive samples were detected, and 78 strains of FAdV were isolated. *Hexon* gene sequences indicated that five FAdV serotypes were prevalent in Shandong Province at different levels. FAdV-11 was the most prevalent serotype, and IBH caused by FAdV-11 is one of the most common current diseases in chicken farms. Although its pathogenicity is relatively low, it inevitably causes greater economic losses when chickens are infected. Interestingly, the FAdV-2 serotype, which accounted for 20.51% of the total, was significantly more abundant, compared with previous flow studies (Cui et al., 2020; Li et al., 2022). The FAdV-2 strain begins to show an epidemic trend in Shandong, and poultry farms may be exposed to the epidemic and pathogenic risk of this serotype in the future, which should be considered. Of note, FAdV-2 and FAdV-11 are unified under the FAdV-D species, which will help identify methods for detecting, preventing, and controlling FAdV-2 serotype infections. The FAdV-4 serotype accounts for 24.36% in the context of universal vaccine immunization in chickens, which has decreased, compared with previous flow data (Li et al., 2022), indicating that immunization and control measures have been effective; although various vaccines (inactivated and attenuated) against FAdV-4 are widely used to immunize all types of flocks, its prevalence remains high and should not be taken lightly (Steer-Cope et al., 2017; Huang et al., 2019; Jiang et al., 2019; Meng et al., 2019; Wang et al., 2020). The prevalence of FAdV-8b remains unabated, and it is worth noting that recombination in FAdV-E may occur, for example, between serotypes FAdV-8a and FAdV-8b (Lv et al., 2021), which is a useful reference for developing control strategies and monitoring the evolution of this type of FAdV in chicken farms. In addition, FAdV-susceptible chickens were 3- to 6-week-old broilers, which is consistent with the findings of a previous study (Niu et al., 2016). The results of the present study will help develop vaccines against multiple adenovirus serotypes to facilitate the control and prevention of FAdV infections in poultry.

FAdV serotypes are diverse. The less pathogenic serotypes are typically subclinical; however, clinical signs may occur upon stimulation by other factors, especially during co-infections, and mortality rates can increase sharply (Jiang et al., 2019). Chickens infected with FAdV-2, FAdV-11, or FAdV-8b may exhibit IBH (Steer et al., 2015; Steer-Cope et al., 2017; Xia et al., 2017). Infection with a highly pathogenic serotype may cause considerable damage to an entire flock. For example, chickens infected with FAdV-4 may exhibit IBH and HPS with a mortality rate of approximately 50% (Wang et al., 2022). In our study, chickens infected with FAdV-4 exhibited IBH and typical HPS, and those infected with FAdV-2, FAdV-8b, and FAdV-11 showed mild IBH symptoms. The FAdV-4-infected group exhibited the highest mortality rate, and the mortality peak coincided with viral shedding and viremia. These peaks occurred earlier and to a greater extent than in the other three groups. In addition, FAdV-4 caused significant histopathological changes and tissue damage due to replication in various tissues, which is consistent with the findings of a previous study (Niu et al., 2019). Although the mortality of FAdV-2 and FAdV-11 is not high, they can replicate in the tissues examined here and cause a partial loss of function, thus causing various degrees of damage to the body. The mortality rate in the FAdV-8b group was only 10%, which was the lowest of all infected groups, and the virus was able to replicate in all examined tissues but showed a low relative viral load, making it less pathogenic, which is consistent with the previous findings (Pan et al., 2020). FAdV was detected in various tissues and excreta of all infected chickens, including blood, heart, liver, spleen, lung, kidney, and bursa, with the highest viral load typically occurring in liver tissue (Niczyporuk and Czekaj, 2018; Abghour et al., 2021), which is in line with our results. Hepatic cells facilitate the replication of FAdV, and CAR receptors on the surface of hepatic cells are the main receptors for FAdV to bind to cells (Tan et al., 2001). The viral load was higher in each tissue of the FAdV-4 infected group than in those of the other three groups, which in part confirms the higher pathogenicity of FAdV-4. FAdV-4 is shorter than other serotypes of *fiber* and readily binds to and interacts with the D2 structural domain of the hepatocyte CAR receptor, facilitating viral invasion (Pan et al., 2020). FAdV-4 not only affects its pathogenicity from viral invasion and replication but also suppresses the normal function of host immunity.

FAdV control currently relies on vaccine immunization; however, there are monovalent and multivalent vaccines with different serotypes of vaccine strains, and the decision of which serotype vaccine to administer may be complicated. The epidemiological findings of the present study may provide a reference for farmers in their choice of vaccine. However, further studies are needed to elucidate the causes underlying the differences in pathogenicity of the various FAdV serotypes so as to facilitate the development of adapted vaccines and examine pathogenesis.

## 5. Conclusion

In summary, our results showed that FAdV-1, FAdV-2, FAdV-4, FAdV-8b, and FAdV-11 were prevalent in Shandong Province to varying degrees. The pathogenicity experiment confirmed that FAdV-4 is the most pathogenic serotype, causing the typical pericardial effusion–inclusion hepatitis syndrome. It seriously threatens the healthy development of the chicken breeding industry. The current prevention and control of the disease are mainly dependent on vaccines. The prevalent strains are changing every year, making the development of a vaccine with good cross-protection particularly important. Good feeding management, timely disease monitoring, and strict biosecurity measures remain the key to the prevention and control of disease on farms.

## Data availability statement

The datasets presented in this study can be found in online repositories. The names of the repository/repositories and accession number(s) can be found in the article/supplementary material.

## Ethics statement

The animal study was reviewed and approved by the Animal Ethics Committee of Shandong Agricultural University.

## Author contributions

TW and FM: writing—original draft preparation. BL, FM, and ML: writing—review and editing. TW and ML: software. CC, YS, PL, JX, ZF, XQ, and FW: investigation. All authors have read and agreed to the published version of the manuscript.
